# Advances in Zr-mediated radical transformations and applications to total synthesis

**DOI:** 10.3762/bjoc.22.3

**Published:** 2026-01-05

**Authors:** Hiroshige Ogawa, Hugh Nakamura

**Affiliations:** 1 The Hong Kong University of Science and Technology (HKUST), Clear Water Bay, New Territories, Hong Kong SAR, China

**Keywords:** halogen atom transfer, photoredox, radical, total synthesis, zirconium

## Abstract

Radical reactions, which have been reported in large numbers in recent years, have exerted major influence across fields where organic synthesis plays a central role, including pharmaceuticals, agrochemicals, materials chemistry, organic semiconductors, and organic thin-film solar cells. These areas are intimately linked to human life; thus, advances in organic synthesis are essential for improving human well-being. Nearly two centuries after the seminal 1828 synthesis of urea from inorganic precursors – often regarded as the birth of organic synthesis – the field continues to evolve rapidly and to exert profound impact on society. A retrospective of almost 200 years of organic synthesis shows that the development and discovery of two-electron ionic transformations dominated the early stages. Over time, pericyclic reactions exemplified by the Woodward–Hoffmann rules and one-electron radical processes became prominent research topics. Today, many of these classical transformations have been further refined to afford reactions that are cheaper, safer, and less toxic. In this context, we focus on mild radical reactions mediated by zirconium (Zr), which has recently attracted attention because of its low toxicity and ease of handling. We discuss the utility of Zr in such radical processes and consider prospects for future development.

## Introduction

Zirconium, a transition metal in the same group as titanium, has been employed across research fields and in medical applications owing to its distinctive physical and chemical properties [1]. Among its notable physical attributes is its high corrosion resistance: metallic zirconium is exceptionally stable toward acids and bases at ambient temperature and is less susceptible to corrosion than titanium. Its low toxicity and excellent biocompatibility have historically supported the use of zirconium in jewelry and in metal trading for investment. As an oxide, zirconium dioxide (ZrO_2_) is also widely used as a white pigment in cosmetics and as a material for artificial teeth, reflecting its long-standing presence in everyday life.

**Figure 1:**
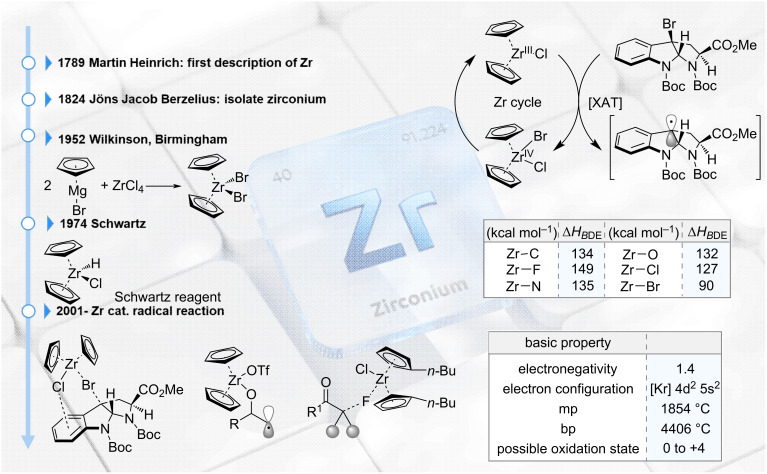
Historical background of zirconium and its physical properties. Image depicted in the background of Figure 1 was purchased from iStock.com/Just_Super. This content is not subject to CC BY 4.0.

Regarding supply, zirconium is typically obtained from silicate minerals (ZrSiO_4_) in igneous rocks, which are widely distributed in the Earth's crust. Consequently, compared with certain rare metals whose sources are limited – such as iridium, rhodium, palladium, platinum, and ruthenium – zirconium is relatively inexpensive and readily available. Zirconium was first discovered by Klaproth in 1789 and subsequently isolated by Berzelius in 1824. In 1952, Wilkinson and Birmingham synthesized zirconium-containing organometallic compounds, pioneering research in organozirconium chemistry [2]. In 1974, Schwartz et al. reported the Schwartz reagent, which remains widely used today as a reducing agent [3] (Figure 1). In recent years, this accessibility, together with advances in radical chemistry, has motivated many researchers to investigate control of radical reactions using zirconium.

A particularly important physical property of zirconium is its large bond dissociation energy (BDE). Although zirconium shares many characteristics with titanium as a group-congener transition metal, it is known to form stronger bonds in several cases. For example, the Ti–O BDE is reported as 115 kcal·mol^−1^, whereas the Zr–O BDE is 132 kcal·mol^−1^ [4]; likewise, Ti–Br and Zr–Br BDEs are 79 and 90 kcal·mol^−1^, respectively [5]. Despite being a congener of titanium, zirconium exhibits substantially higher BDEs (Figure 1).

Building on this characteristic, a range of one-electron processes mediated by zirconium has been reported in recent years. In 2024, Ota and Yamaguchi published a review on radical reactions catalyzed by zirconium complexes [6]. Their review provides a comprehensive summary, particularly of photo-redox reactions involving zirconium catalysis. In the present review, we focus on three aspects: 1) Zr-mediated stoichiometric radical reactions 2) Zr-catalyzed radical reactions 3) Applications of Zr-mediated reactions in total synthesis. To apply these reactions to total synthesis, high functional group compatibility is required. Thus, the insights gained here can be used to assess the potential of zirconium-catalyzed radical reactions.

## Review

### Zr-mediated stoichiometric radical reactions

**Scheme 1:**
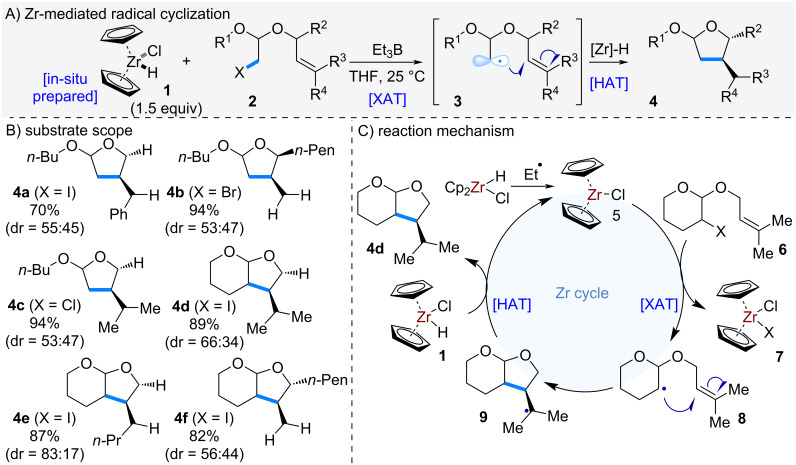
Zr-mediated radical cyclization.

Traditionally, the use of Zr complexes in organic reactions had been limited to two-electron processes; however, the first example of a Zr-mediated radical reaction was reported by Oshima et al. in 2001. They reported an intramolecular radical cyclization using Schwartz’s reagent (Scheme 1) [7–8]. When compound **2**, bearing an alkyl halide and an olefin moiety, was treated with triethylborane and Schwartz’s reagent in tetrahydrofuran, a halogen atom transfer (XAT) occurred at the alkyl halide, generating alkyl radical **3**. This radical subsequently underwent intramolecular addition to the olefin, affording the cyclized acetal product **4**. The reaction proceeded efficiently across a range of substrates bearing various substituents at R¹–R⁴, delivering the desired cyclized products **4a**–**f** in good yields. The proposed mechanism is illustrated in Scheme 1C. Initially, Schwartz’s reagent reacts with triethylborane to generate a low-valent zirconium complex **5**. This complex abstracts the halogen atom from the alkyl halide, forming alkyl radical **8**. The radical then cyclizes onto the olefin, and the resulting radical intermediate undergoes hydrogen atom transfer (HAT) from Schwartz’s reagent to furnish product **4d**.

**Scheme 2:**
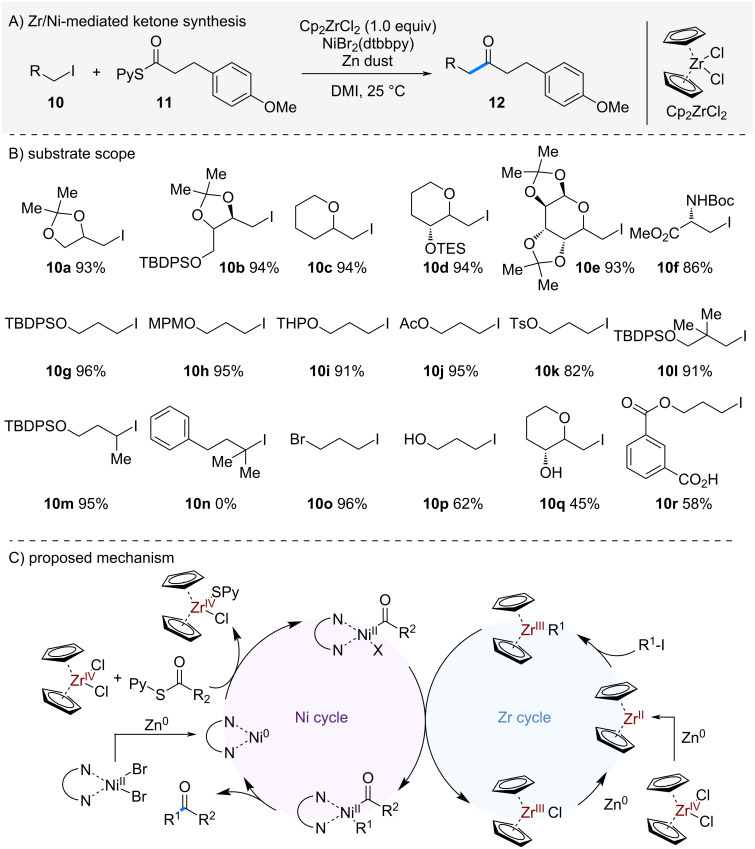
Ni/Zr-mediated one-pot ketone synthesis.

In 2017, Kishi et al. reported a radical-based ketone synthesis employing zirconocene and a nickel catalyst (Scheme 2A) [9]. Traditional anion-based ketone syntheses (e.g., Grignard or RLi reagents) are often unsuitable for complex molecules due to the strongly basic conditions required. As an alternative, dithiane-based ketone synthesis has long been used, but it suffers from drawbacks in terms of step economy [10–11]. To address these limitations, the authors developed a radical-based one-pot ketone synthesis that proceeds under mild conditions.

When thioester **11** and alkyl iodide **10** were subjected to nickel catalysis in the presence of zirconocene dichloride and zinc dust, intermolecular coupling occurred to afford ketone **12** in good yields. This reaction was applicable to substrates bearing β-alkoxy substituents, which are typically problematic under anionic conditions, delivering the desired ketones **12** in good yields. The reaction also tolerated a wide range of protecting groups, including esters, carbamates, and silyl ethers, without degradation (**10f**–**k**). Neopentyl iodide **10l** and secondary alkyl iodide **10m** were also competent substrates, although tertiary alkyl iodide **10n** failed to give the desired products. In substrate **10o** containing both iodide and bromide, selective activation of the alkyl iodide was observed, affording the coupled product in 96% yield. Furthermore, the reaction proceeded smoothly in the presence of alcohols **10p**–**q** and carboxylic acid **10r**, demonstrating excellent functional group tolerance.

The proposed mechanism is shown in Scheme 2C. Zirconocene dichloride is first reduced by zinc dust to generate Zr^II^. The resulting low-valent zirconium species reacts with the alkyl iodide to form a metal-centered radical intermediate. This species undergoes transmetallation with a Ni^II^ complex generated by oxidative addition of the thioester to Ni^0^. Finally, reductive elimination from Ni^II^ furnishes the ketone. Given the strong Zr–S bond (BDE = 137 kcal/mol) [12], zirconocene is thought to play a key role in facilitating the oxidative addition of Ni^0^ to the thioester and accelerating the whole process.

### Zr-mediated catalytic radical reactions

In recent years, numerous chemical transformations employing photoredox catalysts in combination with catalytic amounts of zirconium complexes have been reported. In this section, representative examples of such reactions are summarized and described.

**Scheme 3:**
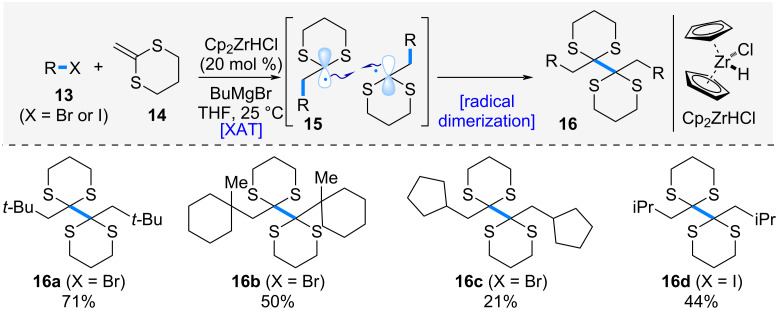
Zirconocene-catalyzed alkylative dimerization of 2-methylene-1,3-dithiane.

In 2007, Oshima et al. reported the zirconocene-catalyzed alkylative dimerization of 2-methylene-1,3-dithiane (Scheme 3) [13]. When alkyl halide **13** was treated with Schwartz’s reagent in the presence of butylmagnesium bromide, the generation of an alkyl radical via XAT process and its subsequent radical addition to 2-methylene-1,3-dithiane (**14**) proceeded, affording radical intermediate **15**. This radical intermediate rapidly underwent homodimerization to give *vic*-bis(dithiane) **16**. The reaction could be applied to both tertiary and secondary alkyl halides, providing the corresponding *vic*-bis(dithiane) derivatives **16a**–**d**.

**Scheme 4:**
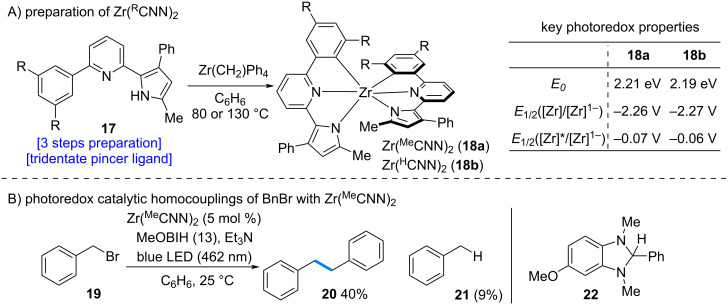
Zirconium complexes as a photoredox catalyst.

In 2018, Milsmann et al. reported the dimerization of benzyl bromide using a zirconium complex as a photosensitizer (Scheme 4) [14]. Heating a zirconium precursor with the pincer-type ligand **17**, which can be synthesized in three steps from commercially available materials, in benzene afforded the Zr(^R^CNN)_2_ complex **18**. Investigation of the photoredox properties of the synthesized complex revealed trends similar to those of known photosensitizers, suggesting its potential utility as a photosensitizer [15]. Accordingly, the Zr(^Me^CNN)_2_ complex was employed as a photosensitizer, together with compound **22** as a sacrificial reductant, in the dimerization of benzyl bromide (**19**). The desired dimerized product **20** was obtained in 40% yield. This study highlights the potential of zirconium complexes as a photoredox catalyst, pointing to promising opportunities for further development in this area.

**Scheme 5:**
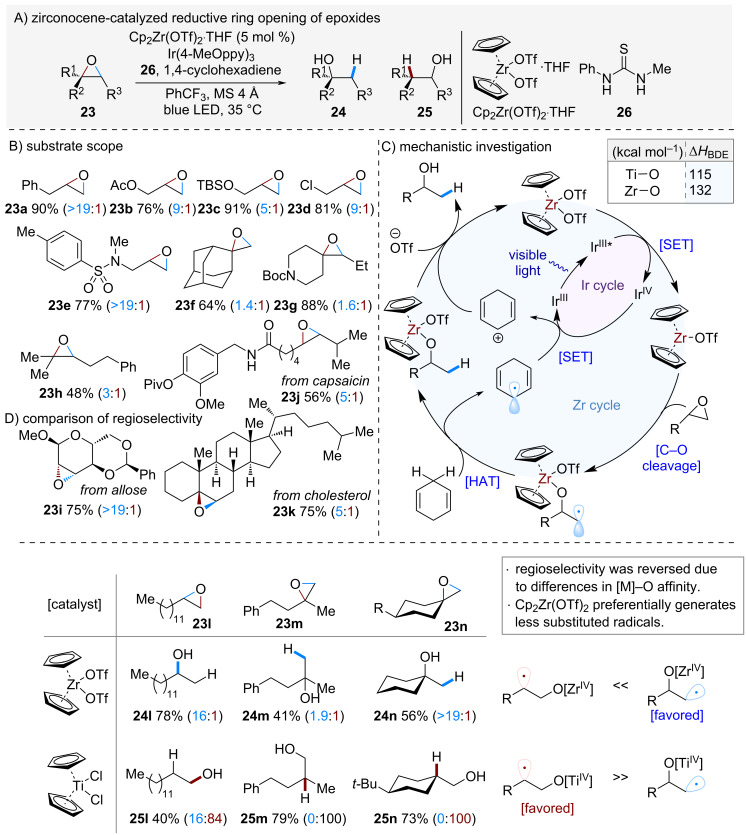
Zr-catalyzed reductive ring opening of epoxides.

In 2022, Ota and Yamaguchi et al. reported the zirconocene-catalyzed ring-opening of epoxides using Cp₂Zr(OTf)_2_·THF (Scheme 5) [4]. Traditionally, such transformations are carried out with low-valent titanium catalysts, which typically proceed via more substituted alkyl radicals to afford compound **25** as a major product [16–20]. In contrast, this study revealed that the strong affinity of zirconium for oxygen reverses this trend, leading instead to compound **24** as a predominant product.

The generality of the reaction was demonstrated with a variety of unsymmetrical epoxides (Scheme 5B). Both monosubstituted epoxides **23a**–**e** and di- and trisubstituted epoxides **23f**–**h** underwent ring opening with regioselectivity opposite to that observed in conventional systems, affording the corresponding alcohols. Moreover, the method was successfully applied to complex molecules such as allose (**23i**) and cholesterol (**23k**), delivering the desired alcohols in good yields with excellent regioselectivity.

The proposed mechanism is shown in Scheme 5C. Homolytic cleavage of the C–O bond in the epoxide generates a strong Zr–O bond, while the resulting alkyl radical abstracts a hydrogen atom from 1,4-cyclohexadiene to furnish the alcohol product. The active species Cp_2_Zr^III^(OTf) is then regenerated via single-electron transfer (SET) from the Ir photocatalyst.

Notably, the addition of thiourea **26** significantly improved the regioselectivity in this reaction. According to NMR experiments, coordination of thiourea **26** to the Zr center was suggested. This effect is considered to have tuned the reactivity of the Zr-catalyst, thereby having a positive effect on the reaction process.

Finally, the regioselectivity of epoxide ring opening was compared between low-valent titanocene and zirconocene catalysts using substrates **23l**–**n**. In both cases, the regioselectivity was found to be reversed. With titanocene, the reaction proceeds through the more stable, substituted radical, whereas with zirconocene, the high oxygen affinity of zirconium is the key factor driving the opposite regioselectivity.

**Scheme 6:**
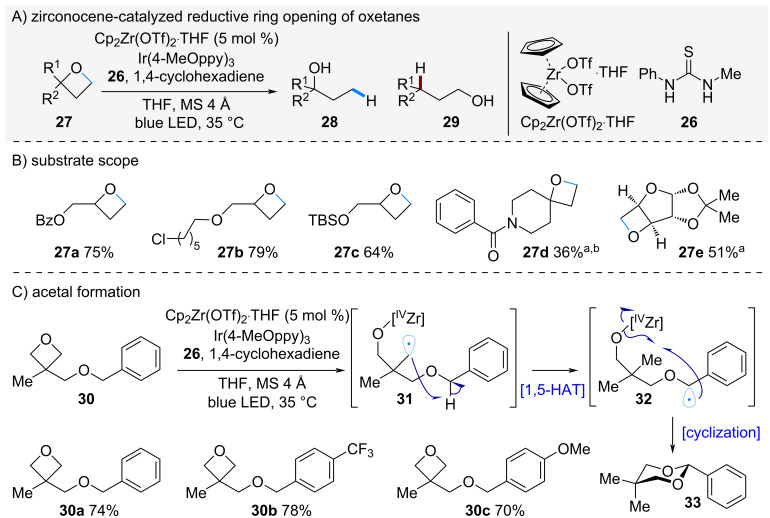
Zr-catalyzed reductive ring opening of oxetanes. ^a^10 mol % of Cp_2_Zr(OTf)_2_·THF was used. ^b^PhCF_3_ was used as a solvent.

In a related study, Ota and Yamaguchi et al. reported the regioselective ring-opening of oxetanes using zirconocene catalysis (Scheme 6) [21]. Treatment of oxetane **27** with zirconocene in the presence of an Ir-photoredox catalyst led to ring opening via the less substituted radical intermediate, affording the corresponding alcohol **28**. In contrast, when substrates bearing a benzylic alcohol moiety at the α-position were subjected to the same conditions, the reaction proceeded through radical intermediate **31**, which underwent a 1,5-hydrogen atom transfer (1,5-HAT) followed by intramolecular C–O bond formation to give the cyclic acetal **33**. This transformation was applicable to a range of oxetanes **30a**–**c** bearing benzylic alcohol derivatives, each affording the corresponding cyclic acetals in good yields.

**Scheme 7:**
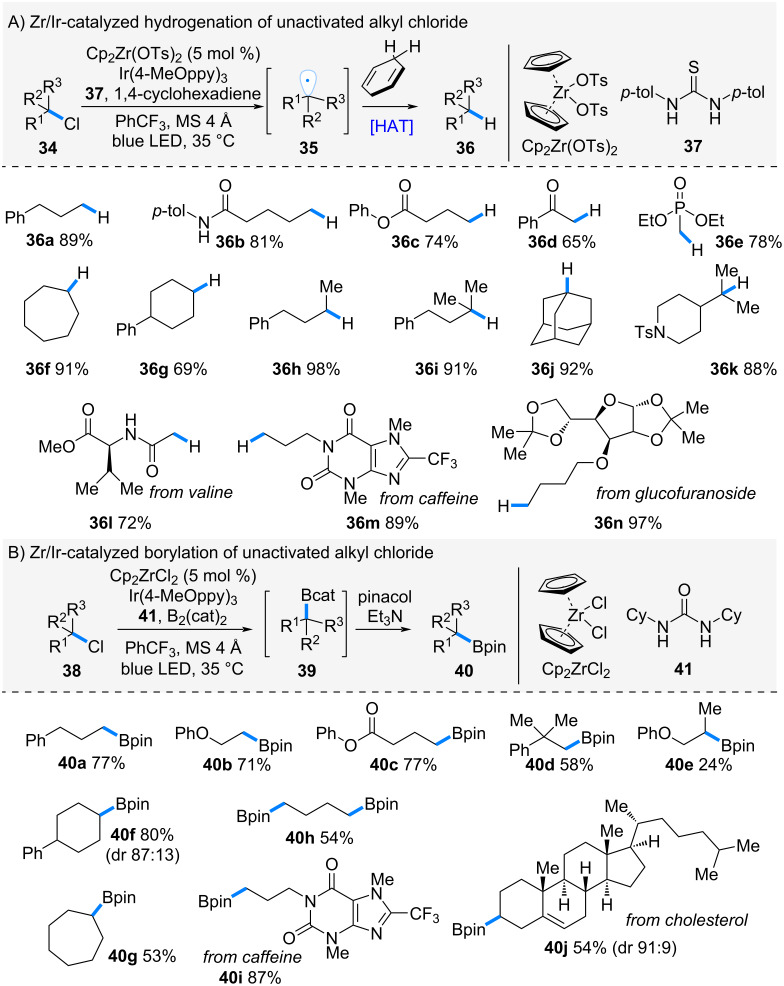
Zr-catalyzed halogen atom transfer of alkyl chlorides.

In 2023, Ota and Yamaguchi et al. reported the hydrogenation of alkyl chlorides via halogen atom transfer (XAT) mediated by zirconocene bistosylate (Scheme 7A) [22]. Alkyl chlorides are inexpensive and readily available feedstocks; however, their chemical transformation has been challenging due to the high bond dissociation energy of the C–Cl bond compared to the C–Br and C–I bonds [12]. Motivated by the strong affinity of zirconium for halogens, they developed a catalytic system to address this issue. Treatment of alkyl chloride **34** with zirconocene bistosylate in the presence of an Ir-photoredox catalyst promoted halogen atom transfer to generate alkyl radical **35**. This radical then abstracted a hydrogen atom from 1,4-cyclohexadiene, affording the hydrogenated product **36**. The reaction proved broadly applicable to a wide range of alkyl chlorides.

For example, primary alkyl chlorides bearing functional groups such as amides (**34b**), esters (**34c**), ketones (**34d**), and phosphoesters (**34e**) underwent smooth hydrogenation to give the corresponding alkanes in good yields. Secondary (**34f**–**h**) and tertiary alkyl chlorides (**34i**–**k**) also reacted smoothly. Moreover, the method was successfully applied to complex molecules, including valine (**34l**), caffeine (**34m**), and glucofuranoside (**34n**), giving the desired products in good yields.

The catalytic system was further extended to the borylation of alkyl chlorides. (Scheme 7B). Under XAT conditions with B_2_(cat)_2_, the in situ-generated alkyl radical was trapped by diborane compound to form intermediate **39**, which was subsequently converted into pinacol boronate **40** upon treatment with pinacol in the presence of Et_3_N. This transformation was likewise applicable to a broad range of alkyl chlorides, affording the corresponding alkylboronates **40a**–**j** in good yields.

**Scheme 8:**
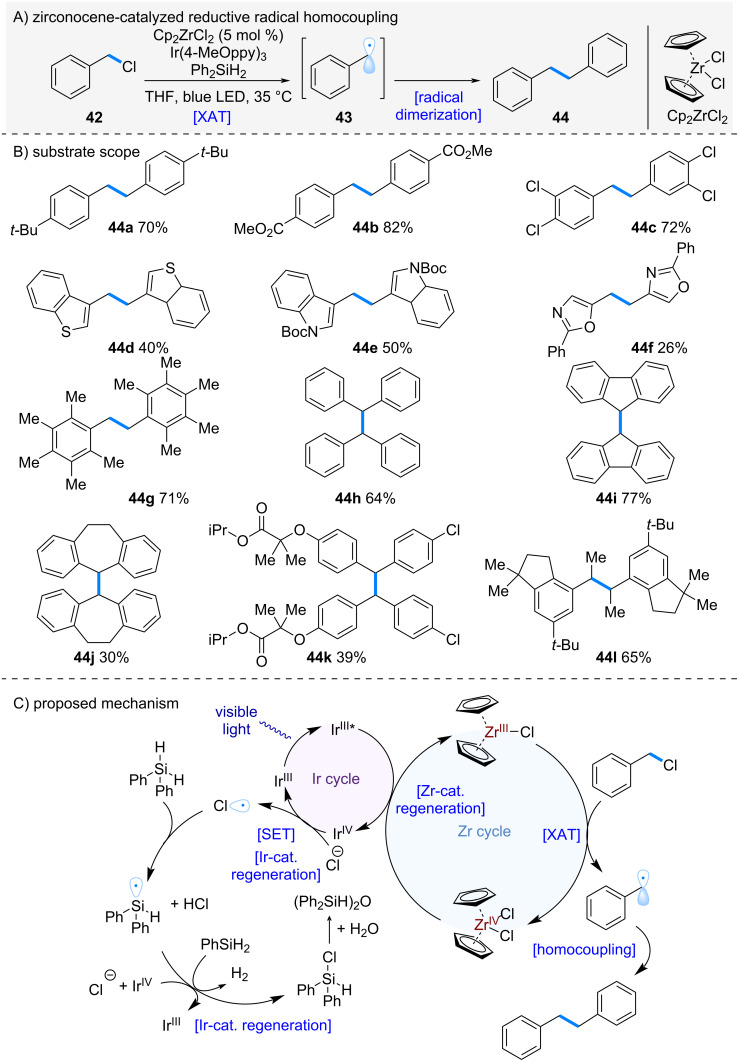
Zr-catalyzed radical homo coupling of alkyl chlorides.

The bibenzyl skeleton is commonly found in numerous natural products and bioactive compounds [23–25], and the development of efficient synthetic methods for its construction remains in high demand. Benzyl chlorides, which are inexpensive and readily available, have attracted attention as promising feedstocks. Against this background, Ota and Yamaguchi et al. in 2025 reported the dimerization of benzyl chlorides using zirconocene in combination with a photoredox catalyst (Scheme 8A) [26]. Treatment of benzyl chlorides with zirconocene dichloride in the presence of an Ir-photoredox catalyst promoted halogen atom transfer (XAT) from the C–Cl bond to generate benzyl radical **43**. Subsequent radical homocoupling afforded the desired bibenzyl dimer **44**.

The reaction proceeded efficiently regardless of the electronic character of the aromatic ring (Scheme 8B, **44a**–**c**). Furthermore, substrates containing various heteroaromatic motifs such as thiophene (**44d**), indole (**44e**), and oxazole (**44f**) also underwent smooth dimerization. The method applied to secondary alkyl chlorides, both acyclic (**44h**) and cyclic (**44i** and **44j**), as well as to benzyl chlorides derived from bioactive molecules such as fenofibrate (**44k**) and celestoride (**44l**), all of which furnished the corresponding dimers in excellent yields.

The proposed reaction mechanism is shown in Scheme 8C. Upon photoexcitation, the Ir^III^* reduces Zr^IV^ to Zr^III^. The resulting low-valent zirconium species abstracts a chlorine atom from the alkyl chloride, generating a benzyl radical, which undergoes homocoupling to give the desired dimer. The addition of PhSiH₂ was found to be beneficial, serving two roles: (1) scavenging HCl generated in the reaction, and (2) trapping chlorine radicals to form silyl radicals, which are subsequently oxidized by Ir^IV^, thereby regenerating Ir^III^.

**Scheme 9:**
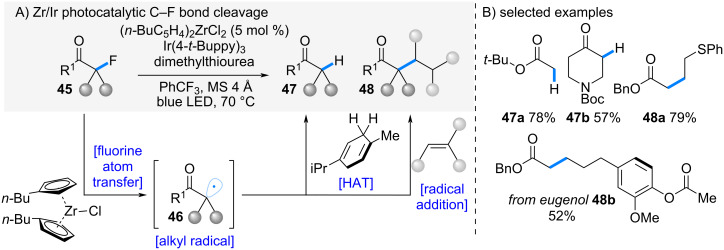
Zr-catalyzed fluorine atom transfer.

In 2024, Ota and Yamaguchi et al*.* reported a zirconocene-mediated defluorination and subsequent functionalization of α-fluorocarbonyl compounds via halogen atom transfer (XAT) (Scheme 9A) [27]. Conventional defluorination reactions had largely been limited to trifluoromethyl groups, which possess relatively positive reduction potentials [28–34]. On the other hand, reductive transformations of monofluoroalkyl groups have been considered difficult due to their negative reduction potentials. In contrast, the authors focused on the comparatively small bond dissociation energy (BDE) of C(sp³)–F bonds. Based on the hypothesis that this property could enable C–F bond functionalization via a halogen‑atom transfer (XAT) mechanism, they initiated the development of monofluoroalkyl group functionalization.

Upon visible-light irradiation in the presence of an Ir photocatalyst and dibutylzirconocene, α-fluorocarbonyl compounds **45** underwent XAT with low-valent zirconium species to generate alkyl radicals **46**. When γ-terpinene was employed as a hydrogen donor, the radicals were reduced to the corresponding alkanes **47**. Alternatively, in the presence of olefins, radical addition proceeded smoothly to furnish functionalized products **48**. This methodology proved broadly applicable to a wide range of α-fluorocarbonyl substrates and olefins (Scheme 9B).

**Scheme 10:**
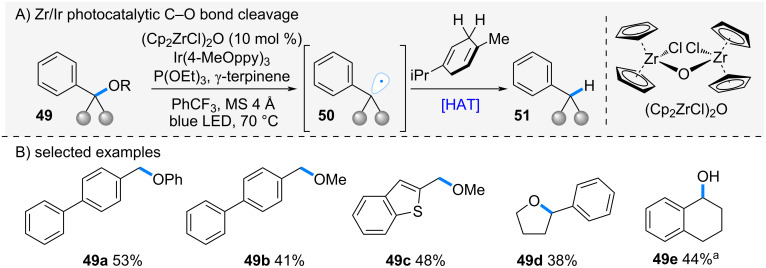
Zr-catalyzed C–O bond cleavage. ^a^Yield without the use of P(OEt)_3_.

In 2025, Ota and Yamaguchi et al. reported a zirconocene-mediated selective cleavage of C–O bonds (Scheme 10A) [35]. The authors had previously demonstrated regioselective ring-opening reactions of epoxides and oxetanes by exploiting the strong affinity of zirconium for oxygen atoms [4,21]. Building on this concept, they envisioned that a similar reaction system could be applied to the homolytic cleavage of C–O bonds in alcohols and ethers.

When benzyl alcohol and ether derivatives **49** were treated with zirconocene in the presence of a photoredox catalyst, homolytic C–O bond cleavage occurred, generating alkyl radicals **50**. These radicals abstracted a hydrogen atom from γ-terpinene, affording the corresponding alkanes. In this reaction, oxo-bridged dimeric zirconocene was employed. This catalytic system was first reported by Romero and co-workers and used for ketone reduction to alcohols [36]. The reaction was applicable to a range of benzyl alcohols and ethers **49a**–**e**, delivering the desired alkanes in moderate yields. Furthermore, when substrates containing multiple C–O bonds **49d** were employed, the reaction proceeded selectively at the benzylic position, giving the hydrogenated products with high site selectivity.

### Zr-catalyzed radical reaction in the total synthesis of complex natural products

This section presents examples of zirconium-mediated reactions applied to the synthesis of complex molecules. For application in total synthesis, high functional group tolerance and mild reaction conditions are required. Accordingly, this section may provide helpful insight into the potential of zirconium-complex-mediated chemical transformations.

**Scheme 11:**
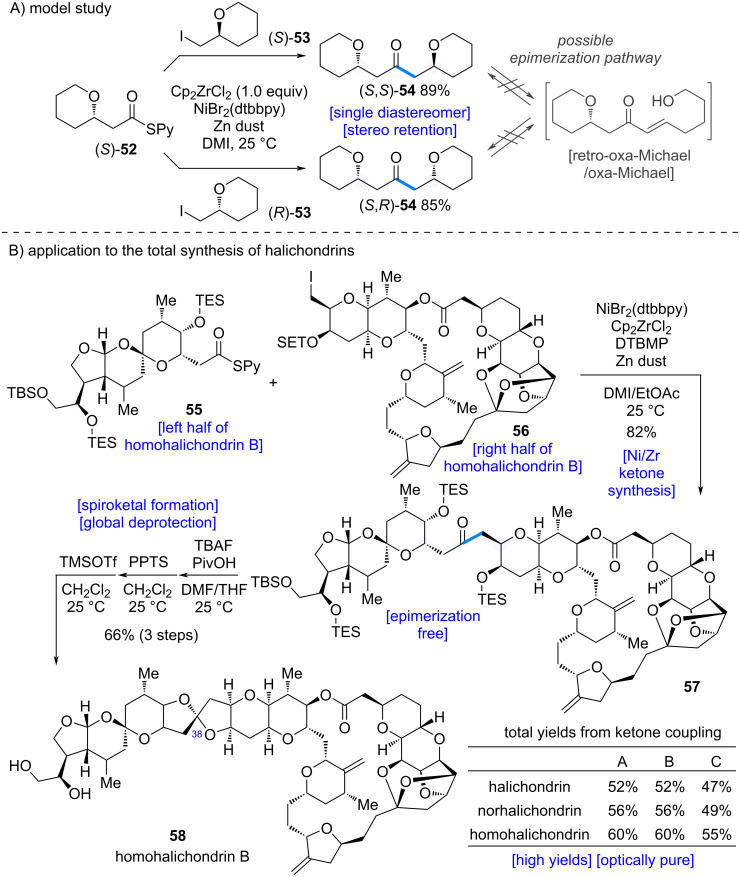
Application to the total synthesis of halichondrins.

Kishi et al. explored the application of their developed ketone synthesis (Scheme 2) [9] to the total synthesis of the halichondrin family of natural products (Scheme 11) [37–38]. Ketone **57**, an intermediate in the synthesis of halichondrins, bears a β-alkoxy substituent, which raises concerns about potential epimerization via a retro-oxa-Michael/oxa-Michael pathway (Scheme 11A). To address this issue, the authors first examined a model system. When β-alkoxythioesters (*S*)-**52** were coupled with chiral alkyl iodides (*S*)-**53** and (*R*)-**53** under the developed conditions, the desired β-alkoxy ketones **54** were obtained in good yields without epimerization, affording optically pure products. These results suggested that the developed reaction would be applicable to halichondrin synthesis.

The Zr/Ni-mediated ketone synthesis was then applied to the total synthesis of homohalichondrin B (**58**, Scheme 11B). Using a fragment corresponding to homohalichondrin B, the coupling reaction furnished ketone **57** in 82% yield. No epimerization at the β-position was observed, and **57** was obtained as a single product. Subsequent removal of the silyl protecting group under PivOH buffer/TBAF conditions, followed by PPTS treatment, enabled spiroketal formation. Finally, epimerization at C38 with TMSOTf completed the total synthesis of homohalichondrin B (**58**).

This synthetic strategy proved broadly applicable to multiple members of the halichondrin family, enabling the efficient synthesis of nine natural products: halichondrins A–C, norhalichondrins A–C, and homohalichondrins A–C. The Zr/Ni-mediated one-pot ketone synthesis developed by the Kishi group thus offers significant advantages over traditional methods, proceeding under mild conditions with easily prepared starting materials. Its versatility highlights the potential for broad application in drug discovery and the development of new materials.

**Scheme 12:**
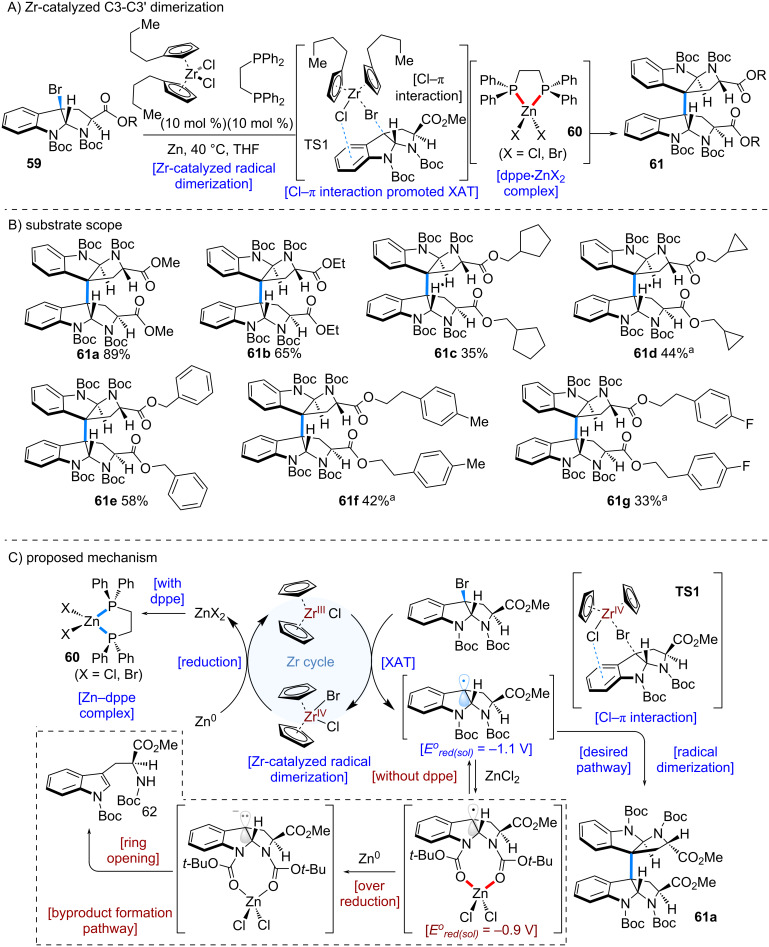
Zr-catalyzed C3 dimerization of 3-bromotryptophan derivatives. ^a^Cp_2_ZrCl_2_ was used.

In 2025, the Nakamura group achieved the first total synthesis of cyctetryptomycins A and B by employing a zirconium catalyst (Scheme 12) [5]. The synthesis commenced with the dimerization of 3-bromotryptophan derivative **59**. As an initial step, the authors sought to develop a mild and scalable method for the dimerization of **59** to afford the dimerized product **61** (Scheme 12A). A screening of various zirconocene catalysts revealed that a sterically bulky dibutylzirconocene catalyst (10 mol %) was particularly effective. Interestingly, the addition of 1,2-bis(diphenylphosphino)ethane (DPPE) (10 mol %) proved to be crucial for the success of the reaction.

DFT calculations suggested that in the transition state (**TS1**), a Cl–π interaction between the chlorine atom on the zirconocene catalyst and the aromatic ring of the substrate facilitated rapid halogen atom transfer (XAT), generating a radical at the C3 position of tryptophan. The resulting radical species underwent fast dimerization to furnish the desired dimer **61**.

This zirconocene-catalyzed dimerization proceeded smoothly under remarkably mild conditions at room temperature, suggesting broad applicability. Indeed, the reaction was shown to tolerate a wide substrate scope, with various 3-bromotryptophan derivatives **59** successfully undergoing dimerization (Scheme 12B).

The proposed mechanism is outlined in Scheme 12C. Zirconocene(IV) is reduced by Zn dust to generate Zr^III^, which then promotes halogen atom transfer from the 3-bromotryptophan derivative **59** to form a radical species. Radical dimerization subsequently affords the desired product **61a**.

Mechanistic studies also revealed that ZnCl_2_, a byproduct of the catalytic cycle, exerts a detrimental effect on the reaction. Specifically, ZnCl_2_ coordinates with the two Boc groups of the substrate, raising the reduction potential from −1.1 V to −0.9 V. This shift increases the susceptibility of the benzylic radical intermediate to over-reduction, leading to undesired anionic species. To address this issue, DPPE was found to be effective: it captures ZnCl_2_ to form complex **60**, thereby suppressing the increase in reduction potential and stabilizing the radical pathway.

**Scheme 13:**
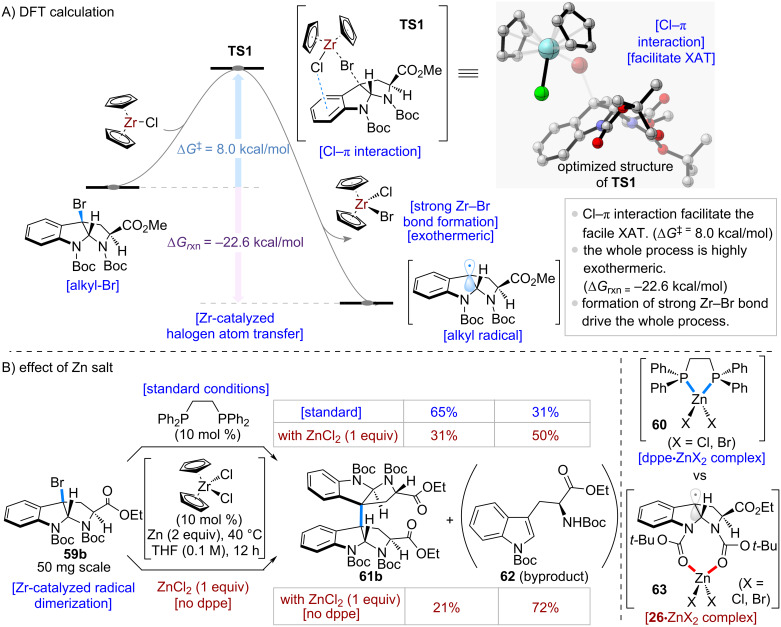
Mechanistic studies.

The DFT calculations are shown in Scheme 13A. The activation barrier for the XAT process was calculated to be Δ*G*^‡^ = 8.0 kcal/mol, indicating that this process can proceed spontaneously around room temperature. Moreover, the transition state (**TS1**) suggested the presence of a Cl–π interaction, which facilitates the facile XAT process. Since this step is accompanied by the formation of a strong Zr–Br bond [12], the overall process is exothermic (Δ*G*_rxn_ = −22.6 kcal/mol) and provides the thermodynamic driving force for the reaction.

Next, control experiments were conducted to evaluate the effect of Zn salts on the reaction (Scheme 13B). The standard dimerization conditions were applied to 3-bromotryptophan derivative **59b**, providing the desired dimer **61b** in 65% yield. The addition of 1 equivalent of ZnCl_2_ reduced the yield to 31%. Furthermore, when ZnCl_2_ (1 equiv) was added in the absence of dppe, the yield dropped further to 21%, and the over-reduced byproduct **62** was obtained in 72% yield. These results suggest that ZnCl_2_ promotes the formation of the over-reduced compound **62**. Notably, the transformation does not require specialized equipment, such as a glovebox, and can be performed on a 100 g scale without difficulty. From a practical standpoint, this feature highlights the method's utility compared to existing approaches.

**Scheme 14:**
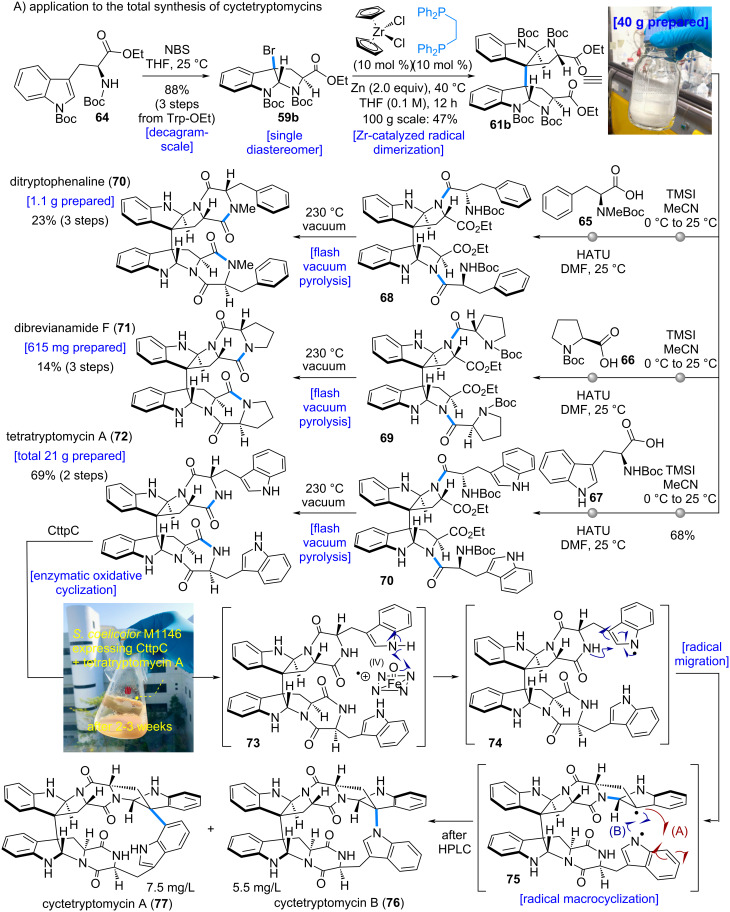
Application to the total synthesis of cyctetryptomycins. A photo of compound **61b** was taken by the authors (Ogawa, H. and Nakamura, H.). This content is not subject to CC BY 4.0. A photo of “S. coelicolor M1146 expressing CttpC + tetratryptomycin A after 2–3 weeks” was reproduced from [5] (© 2024 L. Yu et al., published by Wiley-VCH GmbH, distributed under the terms of the Creative Commons Attribution 4.0 International License, https://creativecommons.org/licenses/by/4.0/

The established Zr-catalyzed dimerization was next applied to the total synthesis of the cyctetryptomycins (Scheme 14, **76** and **77**). The developed dimerization protocol proved scalable to 100 g scale, affording 40 g of the desired dimer **61b** in 47% yield. Subsequent removal of the two Boc groups with TMSI, followed by HATU-mediated amide coupling with various amino acids **65**–**67**, furnished the diketopiperazine precursors **68**–**70**. Heating these precursors under flash vacuum pyrolysis conditions at 230 °C led to the removal of the Boc group and intramolecular dehydration–condensation, thereby constructing the diketopiperazine core and enabling the total syntheses of ditryptophenaline (**70**), dibrevianamide F (**71**), and tetratryptomycin A (**72**).

Notably, this strategy enabled the preparation of tetratryptomycin A (**72**) on a multigram scale, providing a total of 21 g of tetratryptomycin A (**72**).

The synthesized tetratryptomycin A (**72**) was then employed in the total synthesis of cyctetryptomycins (**76** and **77**). Under chemical oxidation conditions, undesired over-oxidation occurred, and no cyclized product was detected. In contrast, enzymatic oxidation using CttpC successfully promoted the desired transformation, affording a separable mixture of cyctetryptomycin A (**76**) and cyctetryptomycin B (**77**). In this way, the total synthesis of the cyctetryptomycins (**76** and **77**) was accomplished. The development of a practical and scalable dimerization method of C3 bromo tryptophan derivative was crucial for this total synthesis.

## Conclusion

Since the first organozirconium reagent was synthesized by Wilkinson and Birmingham in 1954 [2], zirconium has been widely employed in the field of organic synthesis. This widespread use is underpinned by zirconium’s abundance in the Earth’s crust and its low toxicity, among other advantageous properties.

Traditionally, its applications have predominantly involved two-electron processes via hydrozirconation. In recent years, however, single-electron transformations mediated by zirconium complexes have begun to emerge. This review systematically highlights the groundbreaking radical mechanisms involving zirconium complexes that have been reported. Zirconium exhibits unique characteristics – such as a strong affinity for heteroatoms – that offer the potential to enable chemical transformations previously unsolved.

Nevertheless, compared to other transition metals like nickel and palladium, the development of zirconium-based catalysis is still in its early stages, leaving ample room for further exploration. The application of zirconium catalysts in radical reactions is expected to make significant contributions to diverse fields, including drug discovery and the development of advanced functional materials. This review will provide a conceptual foundation for future research in this promising area.

## Data Availability

Data sharing is not applicable as no new data was generated or analyzed in this study.
